# Hypothyroidism-Associated Dyslipidemia: Potential Molecular Mechanisms Leading to NAFLD

**DOI:** 10.3390/ijms222312797

**Published:** 2021-11-26

**Authors:** Maria Mavromati, François R. Jornayvaz

**Affiliations:** 1 Service of Endocrinology, Diabetes, Nutrition and Therapeutic Patient Education, Geneva University Hospitals, 1211 Geneva, Switzerland; maria.mavromati@hcuge.ch; 2 Diabetes Center of the Faculty of Medicine, University of Geneva, 1211 Geneva, Switzerland

**Keywords:** NAFLD, insulin resistance, hypothyroidism

## Abstract

Thyroid hormones control lipid metabolism by exhibiting specific effects on the liver and adipose tissue in a coordinated manner. Different diseases of the thyroid gland can result in hypothyroidism. Hypothyroidism is frequently associated with dyslipidemia. Hypothyroidism-associated dyslipidemia subsequently results in intrahepatic accumulation of fat, leading to nonalcoholic fatty liver disease (NAFLD), which leads to the development of hepatic insulin resistance. The prevalence of NAFLD in the western world is increasing, and evidence of its association with hypothyroidism is accumulating. Since hypothyroidism has been identified as a modifiable risk factor of NAFLD and recent data provides evidence that selective thyroid hormone receptor β (THR-β) agonists are effective in the treatment of dyslipidemia and NAFLD, interest in potential therapeutic options for NAFLD targeting these receptors is growing. In this review, we summarize current knowledge regarding clinical and molecular data exploring the association of hypothyroidism, dyslipidemia and NAFLD.

## 1. Introduction

Nonalcoholic fatty liver disease (NAFLD) is the most important chronic liver disease in the western world, affecting almost 30% of the general population. Moreover, the prevalence of NAFLD can be higher in type 2 diabetic patients and obese patients, affecting up to 90% of people with a body mass index higher than 40 kg/m^2^. NAFLD is also the most rapidly increasing cause of hepatic cirrhosis requiring hepatic transplantation in the future. The pathophysiology of NAFLD is complex and involves multiple hits, but the principal contributing factor to its development is hepatic lipid accumulation, which leads to hepatic insulin resistance [1]. All lipids are not equivalent in terms of their association with the development of insulin resistance. For instance, triglycerides are usually considered inert, whereas diacylglycerols and ceramides can alter insulin signaling [2].

Hypothyroidism can be the result of different diseases of the thyroid gland. Hypothyroidism can be primary, i.e., due to a thyroid gland disorder, or secondary, i.e., due to hypothalamic or pituitary disorders. Primary hypothyroidism is the most frequently encountered in the clinic and can be due to rare congenital disorders (such as thyroid dysgenesis, defective embryonic formation of the gland and genetic diseases) or acquired secondary to different types of thyroiditis (such as Hashimoto’s thyroiditis, silent thyroiditis, subacute thyroiditis and drug-induced thyroiditis) or secondary to surgery or radiotherapy. As thyroid hormones regulate lipid metabolism at various levels in the liver and adipose tissue, hypothyroidism can result in dyslipidemia, which is frequently encountered in hypothyroid patients at the clinic. Dyslipidemia is itself frequently associated with NAFLD. Therefore, NAFLD can be the result of hypothyroidism-associated dyslipidemia. As such, hypothyroidism has been identified as a potentially modifiable factor of NAFLD, and potential therapeutic targets have been identified for the treatment of hypothyroidism-associated NAFLD.

This review aims to present the various potential molecular mechanisms underlying the association between hypothyroidism-related dyslipidemia and NAFLD and clinical data, with a focus on future therapeutic perspectives.

## 2. Thyroid Hormones and Thyroid Hormone Receptors

Thyroid hormones (TH) regulate tissue and cellular metabolism. Tri-iodothyronine (T3) controls gene expression by binding to its receptors. Thyroid hormone receptors (THR) are nuclear receptors, functioning as transcription factors after activation by their ligands [3]. Thyroid hormone receptor isoforms exhibit a tissue-specific expression pattern and function. THR-α (whose gene is located in chromosome 19) mediates TH actions in the heart and brown adipose tissue, whereas, THR-β (whose gene is located in chromosome 3) mediates TH actions on thyroid-stimulating hormone (TSH) secretion and cholesterol metabolism. THR-β has two isoforms. THR-β1 is mainly found in the liver, brain and the kidney, while THR-β2 is found in the hypothalamus and pituitary, exhibiting an important role in the negative feedback of thyroid hormones on the hypothalamic-pituitary axis [4].

Mutations of the THR-β gene are responsible for thyroid hormone resistance syndrome, characterized by tachycardia and increased TSH and free tetra-iodothyronine (FT4) levels.

Thyroid hormone and thyroid receptor agonist treatments have been shown to effectively decrease hepatic steatosis and circulate free fatty acids (FFA) and triglycerides in animal models [5,6]. Research focuses on the beneficial effects of thyroid hormones on metabolism via their receptors while avoiding undesirable effects of systemic hyperthyroidism, such as arrhythmia and bone and muscle loss. Recently, interventional studies have shown the benefits of levothyroxine supplementation in patients with NAFLD and subclinical hypothyroidism in terms of hepatic fat content and liver enzyme levels [7]. Interestingly, these findings were also reproduced in euthyroid individuals with NAFLD [8].

## 3. Thyroid Hormone Effects on Lipid Metabolism in the Liver and the Adipose Tissue

**Figure 1:**
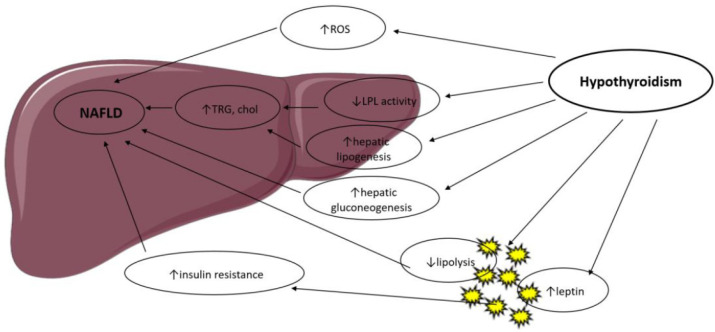
Possible mechanisms in the association between hypothyroidism and NAFLD. LPL: Lipoprotein Lipase; ROS: Reactive Oxygen Species; TRG: Triglyceride; Chol: Cholesterol.

Thyroid hormones control body weight, lipid and carbohydrate metabolism and thermogenesis. They regulate lipid metabolism by exhibiting specific effects on the liver and adipose tissue, summarized in Figure 1, in a coordinated manner but with occasionally contradictory actions [9].

T3 controls the expression of genes involved in hepatic lipogenesis and genes involved in the oxidation of free fatty acids through the thyroid hormone receptor-β, which is the main isoform expressed in the liver [3,10]. Thyroid hormone receptor-α is the main mediator of thyroid hormone actions in the heart and brown adipose tissue. Thus, thyroid hormones regulate lipid metabolism in a tissue-dependent manner, and this was confirmed by studies in knockout mice. THR-α-knockout mice exhibit decreased liver fat content and white adipose tissue mass via a decrease in genes involved in lipogenesis. They have less insulin resistance and hepatic steatosis [11]. THR-β-knockout animals display an increased liver mass and hepatic lipid accumulation through increased lipogenic genes and decreased fatty acid β-oxidation but no significant change in white adipose tissue [11].

Hyperthyroidism has been shown to increase adipose tissue lipolysis [12] and hepatic lipogenesis and is associated with lower body weight, notably due to increased catabolism [13]. These actions are mediated by a T3-induced increase in the expression of several lipogenic genes (such as acyl-CoA-synthetase, fatty acid synthase, acetyl-CoA carboxylase and glucose-6-P dehydrogenase) and genes involved in fatty acid oxidation (such as lipoprotein lipase, fatty acid-binding protein and fatty acid transporter) [3].

Hypothyroidism reduces liver uptake of FFA derived from triglycerides [14] and is associated with a decrease in lipolysis in the adipose tissue and decreased cholesterol clearance [15]. As a result, β-oxidation of free fatty acids and triglyceride clearance is reduced, with a consequent hepatic accumulation of triglycerides and increased low-density lipoprotein (LDL) uptake. Hypothyroidism reduces hepatic lipase activity, which mediates fatty acid oxidation and long-chain fatty acids’ oxidation for energy production. Lipid storage in the liver is further increased by obesity and low resting energy expenditure, both enhanced by hypothyroidism [16,17]. Thyroid hormone treatment in human and murine models reverses hepatic lipase reduced activity.

In the mitochondria, thyroid hormones stimulate carnitine palmitoyltransferase-1a (Cpt1a), the rate-limiting enzyme in fatty-acid oxidation.

Obesity, in both human and animal studies, is found to lead to lipid accumulation in the liver, resulting in fibrosis and cirrhosis. Increased hepatic lipid deposition induces downregulation of several metabolism-related genes, which are dependent of T3 actions [3].

Thyroid hormones are activators of lipogenesis through direct and indirect mechanisms. T3 stimulates enzymes that catalyze several important steps of hepatic fatty acid synthesis, such as acetyl-CoA carboxylase (which catalyzes the carboxylation of acetyl-CoA to malonyl-CoA, the first step of hepatic fatty acid synthesis) and fatty acid synthetase [18]. T3 also induces several transcription factors that participate in de novo lipogenesis, such as carbohydrate responsive element-binding protein (ChREBP), a strong lipogenic regulator [19]. Thyroid-stimulating hormone is also believed to stimulate hepatic lipogenesis through binding with the TSH-receptor expressed at the surface of the hepatocytes, which further leads to stimulation of the peroxisome proliferator-activated receptor-α (PPARα) pathway and activation of sterol regulatory element-binding transcription factor 1 (SREBP-1c) [20,21]. TSH directly increases hepatic gluconeogenesis and decreases phosphorylation of 3-hydroxy-3-methylglutaryl coenzyme A (HMG-CoA) reductase, the main target of statins, thereby inducing hypercholesterolemia [22].

Animal studies have suggested a role of T3 in hepatic mitochondrial turnover, which is altered in nonalcoholic fatty liver disease; thyroid hormones seem to increase mitochondrial biogenesis and mitophagy through nuclear receptors [16]. On the other hand, hepatic steatosis leads to the suppression of T3-dependent genes involved in metabolism in both humans and animal models [4,23].

Thyroid hormone signaling also responds to cross-talk interactions between thyroid receptors and other nuclear receptors sensitive to circulating metabolite levels, such as PPARs and the liver X receptor (LXR) [24]. Alteration of lipophagy, the mechanism of autophagy of lipid droplets, which is an important step of lipid mobilization in the liver, is also believed to participate in NAFLD, and T3 has been shown to stimulate lipophagy in vitro and in vivo [25].

Oxidative stress derived from β-oxidation is thought to contribute to the progression of nonalcoholic steatohepatitis (NASH) to hepatocyte inflammation and liver fibrosis. Hyperthyroidism has been shown to increase oxidative stress, leading to liver cell injury [26], while hypothyroidism lowers oxidative stress levels through a decrease in energy expenditure [27]. Thus, thyroid hormones may contribute to the progression of nonalcoholic fatty liver disease to nonalcoholic steatohepatitis, but the exact pathophysiological mechanisms remain to be clarified.

## 4. Thyroid Hormones and Dyslipidemia

Hypothyroidism is associated with hyperlipidemia through modifications in lipid synthesis, absorption, circulation and metabolism. Thyroid hormones increase cholesterol synthesis by increasing the expression of HMG-CoA reductase in the liver [28]. Thus, hypothyroidism leads to decreased hepatic cholesterol synthesis. However, two additional concomitant mechanisms outweighed this effect. First, there is an increase in gastro-intestinal cholesterol absorption mediated by the Niemann-Pick C1-like 1 protein, the target of the lipid-lowering molecule ezetimibe, in the gut [29]. Second, there is a decrease in cell-surface LDL-cholesterol receptors, possibly via T3-mediated effects on the sterol regulatory element-binding protein-2 (SREBP-2), leading to reduced plasma LDL-cholesterol clearance and increased apo-B lipoproteins [30].

Hypothyroidism also decreases cholesterol excretion and plasma triglyceride clearance, the latter through a decrease in lipoprotein lipase levels [31]. Plasma cholesteryl ester transfer proteins (CETPs), shifting cholesterol from high-density lipoproteins (HDL-C) to LDL-C and very low-density lipoproteins (VLDL) are reduced in hypothyroid states [32].

The combined result of the above changes is an increase in total cholesterol and LDL levels, a slight increase in HDL and triglycerides levels and triglyceride accumulation in the liver, a risk factor for the development of nonalcoholic fatty liver disease [33]. Increased triglyceride accumulation in the liver also contributes to the development of hepatic insulin resistance, another condition linking hypothyroidism with NAFLD, which will be discussed later.

Observational studies confirm that among patients with overt hypothyroidism, 30% have increased total cholesterol and LDL levels, and 90% have dyslipidemia. Furthermore, levothyroxine treatment reverses lipid alterations, with the exception of patients with underlying hyperlipidemia [34].

The effect of subclinical hypothyroidism on lipid levels is less obvious, and the results of clinical studies have been inconsistent. Some observational studies found no difference in lipid levels among subclinical hypothyroid patients and matched controls [35,36], whereas others found significantly higher total cholesterol, triglycerides and LDL-C levels in subclinical hypothyroidism [37,38]. Insulin resistance and smoking are believed to be possible confounding factors since they both induce higher cholesterol increase in the presence of hypothyroidism [34].

## 5. NAFLD and Dysregulated Lipid Metabolism

The pathophysiology of NAFLD is complex, multifactorial and involves multiple systemic alterations [39]. The classical “two-hit” theory is divided into a first “hit” with intrahepatic accumulation of fatty acids and a second “hit” that includes other factors such as oxidative stress and mitochondrial dysfunction. Nevertheless, this theory has been considered inadequate to fully represent the pathogenesis of NAFLD. Therefore, it has been replaced by the “multiple parallel hits” hypothesis that more accurately represents the process of NAFLD development and progression. Indeed, various factors, such as genetic and environmental factors (notably dietary habits), act in parallel and in a synergic way to cause NAFLD [39,40]. NAFLD is due to hepatic lipid accumulation that will subsequently lead to hepatic insulin resistance, alterations in gut microbiota and other deleterious phenomena such as mitochondrial dysfunction, endoplasmic reticulum stress, oxidative stress and production of reactive oxygen species [41]. These different deleterious elements will subsequently lead to a chronic inflammatory state in the liver, promoting NAFLD and NASH [42].

**Figure 2:**
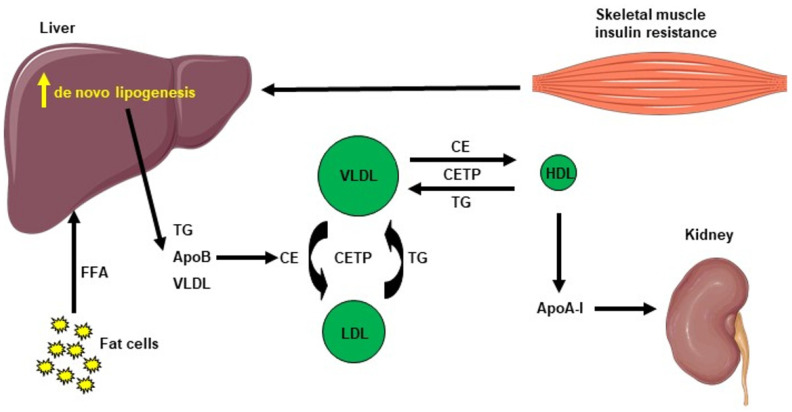
Cholesterol metabolism induced by hepatic de novo lipogenesis. Skeletal muscle insulin resistance increases hepatic de novo lipogenesis, leading to increased hepatic triglycerides (TG). TG can be exchanged for high-density lipoprotein (HDL) cholesterol in the presence of increased plasma very low–density lipoprotein (VLDL) concentrations and normal activity of cholesteryl ester transfer protein (CETP). A VLDL particle then donates a molecule of TG to an HDL particle in return for one of the cholesteryl ester (CE) molecules from HDL. The TG-rich HDL particle can be hydrolyzed of its TG, leading to dissociation of the Apolipoprotein A-1 (Apo A-1) protein. The resulting free Apo A-1 is cleared more rapidly in plasma than the apo A-1 bound to HDL particles, leading to reduced circulating apo A-1, HDL cholesterol and the number of HDL particles.

Hepatic lipid accumulation consists of different lipid intermediates, such as triglycerides, which are usually considered inert, and diacylglycerols and ceramides, which have been shown to cause hepatic insulin resistance in different animal models of nonalcoholic fatty liver disease [43,44,45,46,47,48,49]. Insulin resistance also promotes hepatic de novo lipogenesis and adipose tissue lipolysis, leading to an increased flux of free fatty acids to the liver [50]. This process is also associated with an increase in plasma triglycerides (TG) concentration and a reduction in plasma HDL concentration, contributing to the atherogenic dyslipidemia seen in NAFLD [51]. The plasma HDL level is usually lower in insulin-resistant states, which can be explained by the following mechanism: VLDL TG can be exchanged for HDL cholesterol in the presence of increased plasma VLDL concentrations and the normal activity of cholesteryl ester transfer protein, where a VLDL particle gives a molecule of TG to an HDL particle in return for one of the cholesteryl ester molecules from HDL. This mechanism leads to a cholesterol-rich VLDL remnant particle that is atherogenic and a TG rich, cholesterol-depleted HDL particle [52]. The TG-rich HDL particle will then undergo further change, notably hydrolysis of its TG, which will lead to the dissociation of the apoA-1 protein. Subsequently, the free apoA-1 will be cleared more rapidly in the plasma than the apoA-1 bound to HDL particles, and this process results in reduced circulating apoA-1, HDL cholesterol and the absolute number of HDL particles [53], as summarized in Figure 2. Altogether, these processes lead to the dysregulated lipid metabolism seen in NAFLD.

## 6. Hypothyroidism and NAFLD: Clinical Studies

Insulin resistance, diabetes, obesity and dyslipidemia have all been linked to nonalcoholic fatty liver disease. Hypothyroidism has recently been identified as a potentially modifiable risk factor of NAFLD [54]. Thus, the high prevalence of hypothyroidism, the fact that levothyroxine is a widely available and affordable treatment and recent data providing evidence that THR-b agonists are effective in the treatment of dyslipidemia and NAFLD, all contribute to the growing interest in this association.

Thyroid hormones are regulators of various metabolic processes such as energy expenditure, lipid and glucose homeostasis and body fat distribution, and hypothyroidism is associated with an increased risk of developing components of metabolic syndrome [20]. As a result, several studies have explored the relationship between thyroid status and NAFLD, summarized in Table 1.

The prevalence of NAFLD, diagnosed by ultrasound, in patients with treated hypothyroidism was found to be 30%, compared to 19% in controls, and treated hypothyroidism remained predictive of NAFLD after adjustment of other risk factors, such as age, gender, body mass index (BMI), hypertension and diabetes, with a 1.38 OR (95% CI: 1.17–1.62) [17,18,19,20,21,22,23,24,25,26,27,28,29,30,31,32,33,34,35,36,37,38,39,40,41,42,43,44,45,46,47,48,49,50,51,52,53,54,59]. This relationship is less clear in subclinical hypothyroidism, with some studies concluding that subclinical hypothyroidism represents an independent risk factor for the development of NAFLD and NASH after adjustment for usual confounders [55,56,57,58,61,66]. Other clinical studies failed to prove such an association [62,63,64,67,70].

The inverse relationship also appears to be true. The prevalence of hypothyroidism in patients with biopsy-proven NAFLD was 21% versus 9.5% in controls after adjustment for age, gender, race and BMI [60], and this prevalence seems to be higher in patients with NASH compared with those with a more benign disease [60]. In a meta-analysis including 13,000 individuals, the prevalence of hypothyroidism was around 15–35% among patients with NAFLD, but a clear association was not found [71]. Most cross-sectional and other retrospective studies evaluating this association had inconsistent results, which were also confirmed by two more recent meta-analyses that failed to prove direct causalities [65,68,71,72,73,74]. Some studies have found a strong association between hypothyroidism and NAFLD in a severity-dependent manner [69], while others yielded no association [68]. Nevertheless, a recent systematic review and meta-analysis including 42,000 patients from 13 studies found a high correlation between NAFLD and hypothyroidism, both subclinical and overt, in a severity-dependent manner. However, overt hypothyroidism more significantly correlated with NAFLD than subclinical hypothyroidism. This result was possibly due to the combined effect of low thyroid hormones and high TSH in the liver [75].

Prospective cohorts are more conclusive: A prospective study in a Chinese population suggested that subclinical hypothyroidism is a risk factor for NAFLD [61]. The Rotterdam Study, a large population-based multicenter prospective cohort including 9419 participants with a 10-year follow-up, also showed that low thyroid function is associated with a higher risk of developing NAFLD with a 1.24-fold higher risk of hypothyroidism compared with the euthyroid state [68].

Hypothyroidism was also found to be associated with increased NAFLD activity in some studies but not with fibrosis or steatosis severity [16], while others found a strong association between biopsy-proven advanced fibrosis in NAFLD with increasing TSH levels in a dose-dependent manner, even in the euthyroid range [76]. Furthermore, in a large population-based US study, low thyroid function was found to be an independent predictor of all-cause and cardiovascular mortality in patients with NAFLD [77].

The pathophysiological hypothesis underlying this epidemiological association seems to be the development of insulin resistance in hypothyroidism, probably through increased oxidative stress, lipid peroxidation and the rise of several adipocytokines such as leptin and tumor necrosis factor α (TNFα) [78]. Some advocate that hypothyroidism-induced NAFLD could be a separate clinical entity with specific treatment options [79].

## 7. Thyroid Hormone Analogues for Dyslipidemia Treatment

Although hypothyroidism and NAFLD seem to share at least some common pathophysiological mechanisms, no guidelines exist for the combined treatment of these two entities. Considering the decrease of hepatic lipid accumulation through thyroid hormone actions, the latter could theoretically represent a potential therapeutic option for patients with NAFLD. Thyroid hormone analogues have different affinities with thyroid hormone receptors, leading to diverse biologic effects. Research focused on the development of thyroid receptor agonists with selective hepatic action offers a favorable impact on lipid metabolism without adverse effects on the cardiovascular system. THR-β selective agonists can thus be useful while avoiding the side effects of systemic hyperthyroidism.

The first thyroid receptor analogues were developed and studied in 1990. SKF-94901 and CGS-23425 resulted in a significant decrease in cholesterol levels when administered in animal models. Interestingly, no important side effects were reported. Despite those first promising results, research on both molecules was quickly terminated [80,81].

Studied 20 years ago, GC-1 (sobetirome) and GC-4 were two other analogues with high selectivity for THR-β receptors. Sobetirome binds to TH-β1 receptors with a higher affinity compared to TH-α1 and has tissue-specific accumulation properties, which further enable selectivity [82,83]. Animal studies showed a significant reduction of body weight and adipose tissue. GC-4 is characterized by an inability to cross the blood–brain barrier and, thus, shows no activity in the brain [84].

Eprotirome (KB-2115) is another THR-β agonist developed for the treatment of dyslipidemia. It has a higher affinity for the TH-β receptors in the liver compared to THR-α receptors in the heart. In animal models, eprotirome increased secretion of hepatic cholesterol and inhibited its intestinal absorption. Eprotirome, which also seems to reduce PCSK9, showed a 30–40% reduction of LDL cholesterol in human studies but resulted in liver enzyme increase in some patients [85]. Other changes observed were a reduction in triglyceride, apoB and Lp (a) levels in a dose-related manner. There were no changes in TSH levels and only a slight decrease of T3 and T4 [86,87]. However, the largest multicenter RCT with this agent was stopped prematurely due to cartilage damage in pre-clinical studies in animal models [81].

New generation agents were more focused on the treatment of NAFLD. The agent resmetirom (MGL-3196), used in patients with biopsy-proven NASH in phase 2 trials, resulted in a 30% reduction of LDL cholesterol, 60% reduction of triglycerides and 25% reduction of apoB levels compared to a placebo. The agent also resulted in a significant decrease in intrahepatic fat accumulation, without any change in TSH and free-T3 (FT3) levels and only a slight decrease in FT4 levels, at the highest dose. Heart safety data derived from animal models were very satisfying. This agent was also tested in healthy volunteers, and these studies yielded similar results on lipids and thyroid profile [88,89].

The agent VK-2809 was also used in patients with biopsy-proven NASH in phase 2 trials and induced a significant decrease in intrahepatic fat accumulation after 12 and 36 weeks of treatment [89,90].

3,5-di-iodo-L-thyronine (T2) was also evaluated in animal models with promising results (reduction of lipid levels and fat accumulation) but did not significantly impact lipid profile and insulin resistance in human studies. Thus, despite the absence of cardiac side effects, 3,5-di-iodo-L-thyronine failed to gain further attention. Another thyromimetic, T1AM (3-iodothyronamine), has shown beneficial effects on lipid levels in animal studies but, so far, has not been tested in humans [79].

In 2008, a Californian research group suggested a different approach, which was to develop thyroid hormone analogues that would be selectively transported to the liver, thus, avoiding their actions and side effects in the pituitary–hypothalamic axis and the heart. According to this theory, the agent MB-07811, which is converted to MB-07344 in the liver, was developed and studied. MB-07811 decreases cholesterol plasma levels as well as hepatic steatosis [91,92]. In human studies, MB-07811 reduces LDL cholesterol and triglyceride levels without cardiac side effects compared to a placebo [81].

Selective THR-β agonists have been tested mostly in euthyroid patients. Thus, since THR-β are also found in the pituitary gland, questions on safety and correct monitoring of this treatment in hypothyroid individuals remain. Altogether, resmetirom (MGL-3196) seems to be the most promising agent, although cardiovascular safety data are needed.

## 8. Conclusions

Hypothyroidism is widely prevalent in the general population and is associated with an increased risk of developing components of metabolic syndrome, such as obesity and insulin resistance. Data from clinical studies show increased prevalence of NAFLD in patients with hypothyroidism, and hypothyroidism is more frequent in patients with NAFLD proved by biopsy. Nevertheless, NAFLD is a growing problem in the western world and the most frequent cause of chronic liver disease with complex pathophysiology.

Thyroid hormones (TH) regulate tissue and cellular metabolism, and their receptors exhibit a tissue-specific expression pattern and function. THR-α is mostly found in the heart and brown adipose tissue, while THR-β is found in the liver, brain and the kidney. Thyroid hormones control lipid metabolism in the liver and adipose tissue, and hypothyroidism has been identified as a potentially modifiable risk factor for nonalcoholic fatty liver disease. Hypothyroidism results in increased cholesterol absorption in the gut and decreased LDL cholesterol clearance, leading to higher LDL cholesterol plasma levels and triglycerides accumulation in the liver. Intrahepatic accumulation leads to NAFLD and, subsequently, to the development of hepatic insulin resistance.

Since hypothyroidism and NAFLD seem to share common pathophysiological mechanisms, THR-b selective agonists are developed to provide specific effects in the liver, aiming to reduce fat accumulation while avoiding the side effects of systemic hyperthyroidism and, therefore, represent potential therapeutic molecules targeting NAFLD caused by hypothyroidism-associated dyslipidemia. Several thyromimetics have been studied in the last 30 years, and resmetirom (MGL-3196) seems to be the most promising. Nevertheless, obtaining cardiovascular safety data is a challenging objective for the future.

Translational research could provide a more thorough understanding of the mechanisms underlying tissue-specific actions of thyroid-hormone analogues. Clinical studies need to focus on the beneficial effects on the liver, body weight and lipid levels of long-term treatment with thyromimetics while determining their impact on cardiovascular outcomes and bone density. Therefore, future research on molecular mechanisms linking hypothyroidism-related dyslipidemia and NAFLD development, and clinical data on the association, will provide a better understanding of this complex relationship to provide targeted treatment options.

## Figures and Tables

**Table 1:** Principal clinical studies examining the association between hypothyroidism and NAFLD.

Reference	Study Design	Study Sample	Diagnosis of NAFLD	Definition of Hypothyroidism	Main Findings
Liangpunsakul et al. 2003 [55]	Cross-sectional case-control study	174 patients with NASH and 442 controls	Biopsy (all cases had NASH)	Self-reported use of levothyroxin	Hypothyroidism was independently associated with NASH (OR 2.30, 95% CI 1.20–4.20)
Reddy et al. 2007 [56]	Case-control study	54 patients with HCC of unknown etiology and 2 groups of controls (57 HCC patients with HCV and 49 HCC patients with alcoholic liver disease)	Biopsy or clinical and imaging criteria	TSH > 5 mIU/L, history of hypothyroidism	Hypothyroidism is significantly higher prevalent in subjects with HCC of unknown etiology compared to controls with viral or alcoholic HCC
Silveira et al. 2009 [57]	Cross-sectional study	97 patients with NAFLD	Biopsy	TSH > 5 mIU/L or < 0.3 mIU/L Total T4 > 12.5 μg/dL or < 5 μg/dL History of hyper/hypothyroidism	The prevalence of hypothyroidism in patients with NAFLD was 20%
Xu et al. 2011 [58]	Cross-sectional study	227 patients with NAFLD and 651 controls	Ultrasound	TSH > 4.5 mIU/L or < 0.5 mIU/L FT4 > 14.4 pmol/L or < 7.85 pmol/L	Patients with hypothyroidism are more likely to develop NAFLD (*p* < 0.001), FT4 is a risk factor for NAFLD (OR = 0.847, 95% CI: 0.743–0.966)
Chung et al. 2012 [59]	Cross-sectional study	2324 patients with hypothyroidism and 2324 controls	Ultrasound	Subclinical hypothyroidism: TSH > 4.1 mIU/L and normal FT4 Overt hypothyroidism: TSH > 4.1 mIU/L and FT4 < 0.7 ng/dL	Hypothyroidism is an independent risk factor for NAFLD (OR = 1.38, 95% CI: 1.17–1.67)
Pagadala et al. 2012 [60]	Cross-sectional study	233 patients with NAFLD and 430 controls	Biopsy	Clinical diagnosis and on thyroid replacement therapy	Prevalence of hypothyroidism was higher in NAFLD patients (21.1% vs. 9.5%, *p* < 0.001)
Xu et al. 2012 [61]	Prospective case-control study	327 patients with subclinical hypothyroidism and 327 controls	Ultrasound (15% developed NAFLD after 4.9 years median follow-up)	TSH > 4.5 mIU/L and normal FT4 levels	Subclinical hypothyroidism was independently associated with risk of developing NAFLD (HR 2.21, 95% CI: 1.42–3.44)
Itterman et al. 2012 [62]	Population-based study	3661 individuals without a self-reported history of thyroid or liver disease	Ultrasound (16.1% had NAFLD)	Subclinical hypothyroidism: TSH > 3 mIU/L and normal FT4 Overt hypothyroidism: TSH > 3 mIU/L and FT4 < 7 pmol/L	Hypothyroidism was not independently associated with NAFLD. FT4 levels were inversely associated with NAFLD in men (OR 0.04, 95% CI: 0.01–0.17]) and in women (OR 0.06, 95% CI:0.01–0.42)
Eshraghian et al. 2013 [63]	Cross-sectional study	832 individuals	Ultrasound (15.3% had NAFLD)	Subclinical hypothyroidism: TSH > 5.2 mIU/L and normal FT4 levels Overt hypothyroidism: TSH > 5.2 mIU/L and FT4 < 11.5 pmol/L	Subclinical hypothyroidism was not associated with NAFLD (OR 1.12, 95% CI: 0.51–2.46). Overt hypothyroidism was not associated with NAFLD (OR 0.87, 95% CI: 0.33–2.28)
Posadas-Romero et al. 2014 [64]	Cross-sectional study	753 adults	Computed tomography (31.1% with NAFLD)	Subclinical hypothyroidism: TSH > 4.5 mIU/L and normal FT4	Subclinical hypothyroidism was not associated with NAFLD (OR 0.83, 95% CI: 0.55–1.25)
Lee et al. 2015 [65]	Retrospective cohort study	18,544 individuals	Ultrasound	Subclinical hypothyroidism: TSH > 4.2 mIU/L, normal FT4 Overt hypothyroidism: TSH > 4.2 mIU/L, FT4 < 10.97 ng/dL	NAFLD incidence did not differ significantly with thyroid hormonal status (Subclinical hypothyroidism: HR = 0.965, 95% CI = 0.814–1.143, *p* = 0.67; Overt hypothyroidism group: HR = 1.255, 95% CI = 0.830–1.899, *p* = 0.28)
Parikh et al. 2015 [66]	Case-control study	500 patients with NAFLD and 300 controls	Ultrasound	Subclinical hypothyroidism: TSH > 5.5 IU/mL and <10 IU/mL) Overt hypothyroidism: TSH > 10 IU/mL)	NAFLD was statistically significantly associated with hypothyroidism (OR: 14.94, 95% CI: 3.5–62.6)
Ludwig et al. 2015 [67]	Cross-sectional, population-based study	1276 individuals	Ultrasound (24.7% with NAFLD)	Subclinical hypothyroidism: TSH > 3.4 mIU/L and normal total T4 Overt hypothyroidism: TSH > 3.4 mIU/L and total T4 < 12.8 pmol/L	Hypothyroidism was not associated with NAFLD (OR 1.19 95% CI: 0.65–2.17)
Bano et al. 2016 [68]	Longitudinal prospective cohort study	9419 euthyroid adults	Ultrasound (12.9% developed incident NAFLD after 10 years of median follow-up)	Subclinical hypothyroidism: TSH > 4.0 mIU/L and normal FT4 Overt hypothyroidism: TSH > 4.0 mIU/L and FT4 < 10.9 pmol/L	Hypothyroidism was associated with a 1.24-fold higher NAFLD risk (95% CI: 1.01–1.53). NAFLD risk decreased gradually from hypothyroidism to hyperthyroidism (*p* for trend = 0.003).
Kim et al. 2018 [69]	Cross-sectional study	425 patients with NAFLD	Biopsy	Subclinical hypothyroidism: TSH > 4.5 mIU/L and normal FT4	Subclinical hypothyroidism was independently associated with NASH (OR 1.61, 95% CI: 1.04–2.50) and advanced fibrosis (OR 2.23 95% CI: 1.18–4.23).
Martinez Escude et al. 2020 [70]	Cross-sectional, retrospective population study	10,116 adults	Ultrasound	Subclinical hypothyroidism: TSH > 4.94 UI/mL and normal T4 Overt hypothyroidism: elevated TSH and decreased T4	Hypothyroidism is not associated with NAFLD (*p* = 0.631)

NAFLD: nonalcoholic fatty liver disease, NASH: nonalcoholic steatohepatitis, OR: odds ratio, CI: confidence intervals, HCC: hepatocellular carcinoma.

## Data Availability

All relevant data are within the paper.
